# In vitro effects of sitosterol and sitostanol on mitochondrial respiration in human brown adipocytes, myotubes and hepatocytes

**DOI:** 10.1007/s00394-019-02052-y

**Published:** 2019-07-17

**Authors:** Emmani B. M. Nascimento, Maurice Konings, Gert Schaart, Albert K. Groen, Dieter Lütjohann, Wouter D. van Marken Lichtenbelt, Patrick Schrauwen, Jogchum Plat

**Affiliations:** 1 Department of Nutrition and Movement Sciences, NUTRIM School of Nutrition and Translational Research in Metabolism, Maastricht University, Maastricht, 6200 MD The Netherlands; 2 Department of Vascular Medicine, Amsterdam Diabetes Center, Amsterdam University Medical Center, Amsterdam, 1105 AZ The Netherlands; 3 Department of Pediatrics, University of Groningen, University Medical Center Groningen, Groningen, 9713 ZG The Netherlands; 4 Institute of Clinical Chemistry and Clinical Pharmacology, University Hospital, 53127 Bonn, Germany

**Keywords:** Cellular respiration, Sitosterol, Sitostanol, Brown adipose tissue, Mitochondria

## Abstract

**Purpose:**

Lowering of LDL cholesterol levels by plant sterols and stanols is associated with decreased risk of cardiovascular disease in humans. Plant sterols and stanols also lower triacylglycerol (TG). However, it is not fully understood how reduction in TG is achieved and what the full potential of plant sterols and stanols is on whole-body metabolism. We here hypothesize that high levels of plant sterols and stanols stimulate whole-body energy expenditure, which can be attributed to changes in mitochondrial function of brown adipose tissue (BAT), skeletal muscle and liver.

**Methods:**

Phytosterolemic mice were fed chow diets for 32 weeks to examine whole-body weight gain. In vitro, 24-h incubation were performed in adipocytes derived from human BAT, human myotubes or HepG2 human hepatocytes using sitosterol or sitostanol. Following mitochondrial function was assessed using seahorse bioanalyzer.

**Results:**

Chow feeding in phytosterolemic mice resulted in diminished increase in body weight compared to control mice. In vitro, sitosterol or sitostanol did not change mitochondrial function in adipocytes derived from human BAT or in cultured human myotubes. Interestingly, maximal mitochondrial function in HepG2 human hepatocytes was decreased following sitosterol or sitostanol incubation, however, only when mitochondrial function was assessed in low glucose-containing medium.

**Conclusions:**

Beneficial in vivo effects of plant sterols and stanols on lipid and lipoprotein metabolism are well recognized. Our results indicate that alterations in human mitochondrial function are apparently not involved to explain these beneficial effects.

## Introduction

Dietary plant sterols or stanols lower intestinal cholesterol absorption, which results in lower serum LDL cholesterol. A daily intake of 2-g plant sterols and/or plant stanols lowers serum LDL cholesterol up to 10% [1]. LDL cholesterol is a causal risk factor for the development of cardiovascular disease, thus lowering serum LDL cholesterol would reduce the risk to develop cardiovascular disease. Besides lowering serum LDL cholesterol, plant sterols and plant stanols lower serum triacylglycerol (TG) levels, especially in subjects with elevated serum TG who are at risk to develop metabolic disease [2].

Although the evidence that fasting TGs are independent risk factors for cardiovascular disease is weak [3], accumulating evidence suggests that postprandial TGs are independent risk factors [4, 5]. So far, several studies have demonstrated TG-lowering effects of plant sterols and stanols [6, 7],however, the mechanism behind the TG-lowering effects of plants sterols and stanols remains unclear. In C57Bl/6 mice on a high-fat diet (HFD), both plant sterols and stanols lowered hepatic VLDL production [8], however, the molecular explanation for this effect remains so far unknown. Furthermore, we have demonstrated in humans that plant stanol consumption strongly reduced serum concentration of large TG-rich VLDL in subjects with the metabolic syndrome [9]. Besides reduced hepatic VLDL-1 production, the reduced concentration of VLDL-1 particles could also be explained through enhanced TG clearance from the circulation. Previously, we indirectly excluded a role for LPL-mediated TG uptake in white adipose tissue (WAT) and muscle. No change was observed in circulating concentrations of apoC2 and apoC3 during the postprandial phase, the activator, and inhibitor of LPL, respectively [10]. However, recent evidence suggests that—at least in mice—a considerable fraction of circulating TGs is cleared from the circulation by brown adipose tissue (BAT). BAT, as opposed to white adipose tissue (WAT), is able to combust lipids and glucose as fuel resulting in heat production [11]. Uncoupling protein 1 (UCP1) present in the mitochondria of brown adipocytes, uncouples the proton gradient in the electron transport chain generating heat instead of ATP. Because of this hallmark, BAT has been coined an important target to combat metabolic disease [12, 13]. With respect to lipid metabolism, BAT stimulation via cold exposure in humans specifically showed uptake of the FFA tracer ^18^F-FTHA which was not observed in WAT or muscle [14]. Cold-exposed mice subjected to an oral lipid tolerance test, did not show changes in TG concentrations due to active BAT [15]. Also pharmacological intervention with metformin in mice, lowered circulating levels of TGs via increased VLDL-TG clearance by BAT [16]. Activated BAT might be an important player in lipid metabolism,however, it remains unexplored whether BAT plays a role in the action of plant sterols and stanols. Also the involvement of other organs with high mitochondrial density should not be overlooked. Therefore, we here examined how increasing plant sterols and stanols in mice affected whole-body metabolism in mice. Furthermore, we investigated if the TG-lowering action of plant sterols and plant stanols could be attributed to mitochondrial activity in BAT, skeletal muscle or liver.

## Materials and methods

### Materials

Stock solutions of sitosterol and sitostanol (Sigma) were prepared in ethanol and provided to the different in vitro cell models. Final sitosterol and sitostanol concentrations used were 12 µM and 1.2 µM, respectively, and compared to ethanol as carrier control. All conditions were set at identical ethanol concentration of 0.25%.

### Animal experiments

Male age-matched ApoE × ABCG8 knockout mice and ApoE knockout mice (C57Bl/6J background, in house breeding), were housed in a light (12:12)- and temperature-controlled (21 °C) facility and received laboratory chow (RMH-B, Hope Farms, Woerden, The Netherlands) ad libitum. ApoE × ABCG8 knockout (KO) mice and apoE KO mice were fed chow diet for 32 weeks. Body weight development was monitored by measuring body weight of the mice at weeks 16, 24, 28, and 32. Experiments were conducted in conformity with the law on the welfare of laboratory animals and experimental procedures were approved by the responsible ethics committee of the UMCG (6946). In vivo sterol concentrations were determined as previously described [17].

### Cell culture

HepG2 cells were grown in MEM supplemented with 10% FCS, sodium pyruvate and non-essential amino acids and pen/strep as described [18]. Collection and differentiation of human primary myotubes have been described previously [19]. Growth and differentiation of human primary differentiated adipocytes have been described before [20], however, modifications have been applied to the original protocol. In short, human adipose tissue biopsies derived from deep neck surgery were incubated with collagenase to collect the stromal vascular fraction. Collected cells were grown to confluence before differentiation was initiated. Differentiation was initiated for 7 days via differentiation medium made up of biotin (33 µM), pantothenate (17 µM), insulin (100 nM), dexamethasone (100 nM), IBMX (250 µM), rosiglitazone (5 µM), T3 (2 nM), and transferrin (10 µg/ml). Cells were transferred to maintenance medium consisting of biotin (33 µM), pantothenate (17 µM), insulin (100 nM), dexamethasone (10 nM), T3 (10 nM) and transferrin (10 µg/ml) for 5 additional days.

### Cellular respiration

Cells were plated and/or differentiated in XF96 cell culture microplates (Agilent Technologies, Santa Clara, CA, USA). After 24 h incubation with 12 µM sitosterol or 1.2 µM sitostanol or vehicle, oxygen consumption and mitochondrial function were measured using the Seahorse XF96 extracellular flux analyzer (Agilent Technologies, Santa Clara, CA, USA). Cells were incubated for 1 h at 37 °C in unbuffered XF assay medium. For HepG2, XF medium was supplemented with 5.5 or 25 mM glucose (Sigma Aldrich, Saint Louis, MO, USA), 2 mM GlutaMax (Thermo Fisher Scientific, Waltham, MA, USA) and 1 mM sodium pyruvate (Thermo Fisher Scientific, Waltham, MA, USA). For human primary cultured myotubes XF medium was supplemented with 5.5 mM glucose, 4 mM GlutaMax and 1 mM sodium pyruvate. For cultured human primary adipocytes derived from WAT or BAT XF assay medium was supplemented with 25 mM glucose, 2 mM GlutaMax and 1 mM sodium pyruvate. Basal oxygen consumption rate (OCR) was measured. Followed by injections (which can be seen in the trace) of 1 µM oligomycin (inhibitor ATP-synthase/complex V of the electron transport chain), 1 µM beta-adrenergic agonist norepinephrine (NE), 0.5 µM mitochondrial uncoupler FCCP, 10 mM pyruvate or 1 µM rotenone + antimycin A (inhibitor of complex I and III of the electron transport chain). Oligomycin and NE were purchased from Sigma-Aldrich. In cultured human adipocytes, mitochondrial uncoupling was examined as mitochondrial respiration after the inhibition of ATP synthase with oligomycin (which was set to 100%), thus reflecting mitochondrial uncoupling because of proton leak. In other cultured cells basal respiration was set to 100%.

### Statistics

For XF seahorse cellular respiration experiments, differences were analyzed using a two-way analysis of variance (ANOVA) in GraphPad Prism (GraphPad Software Inc., San Diego, CA, USA). Statistical significance was set at *p* < 0.05.

## Results

**Fig. 1:**
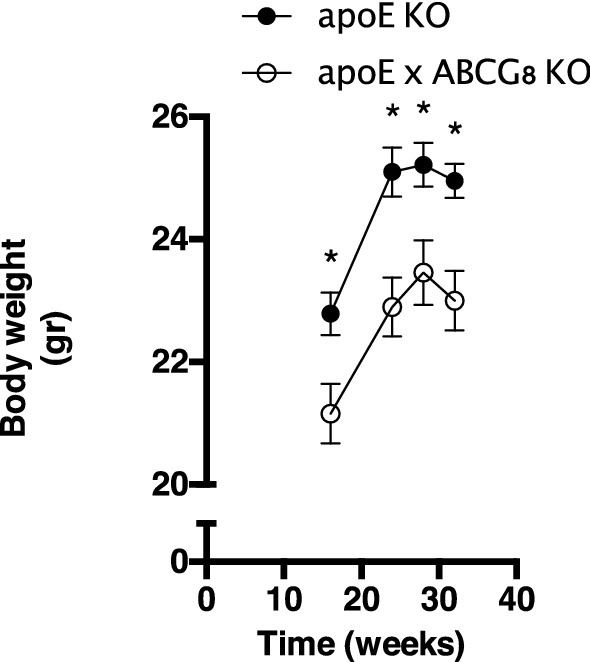
High levels of plant sterol and stanol affect body weight in mice. ApoE KO or apoE × ABCG8 KO were fed a chow diet for 32 weeks. Data are expressed as mean ± SEM (*n* = 7). *p < 0.05

To verify the effects of plant sterols and stanols on whole-body metabolism, body weight development was determined in apoE KO mice and ABCG8 KO mice following a chow diet of 32 weeks. The ABCG8 KO background was chosen because of the manifestation of phytosterolemia [21]. The ABCG8 background increased sitosterol from 4.9 ± 1.1 mg/dl to 67.4 ± 12.7 mg/dl and campesterol from 11.8 ± 2.9 mg/dl to 24.1 ± 4.3 mg/dl (*n* = 14–15)”. As shown in Fig. 1, apoE × ABCG8 KO mice did not increase their body weight in a comparable manner as the apoE KO mice. We here speculate that the reduced body weight development could be explained by increased energy expenditure in organs containing high numbers of mitochondria. Thus to further translate this hypothesis to human tissues we next performed in vitro experiments on human adipocytes, skeletal muscle and liver cells.

**Fig. 2:**
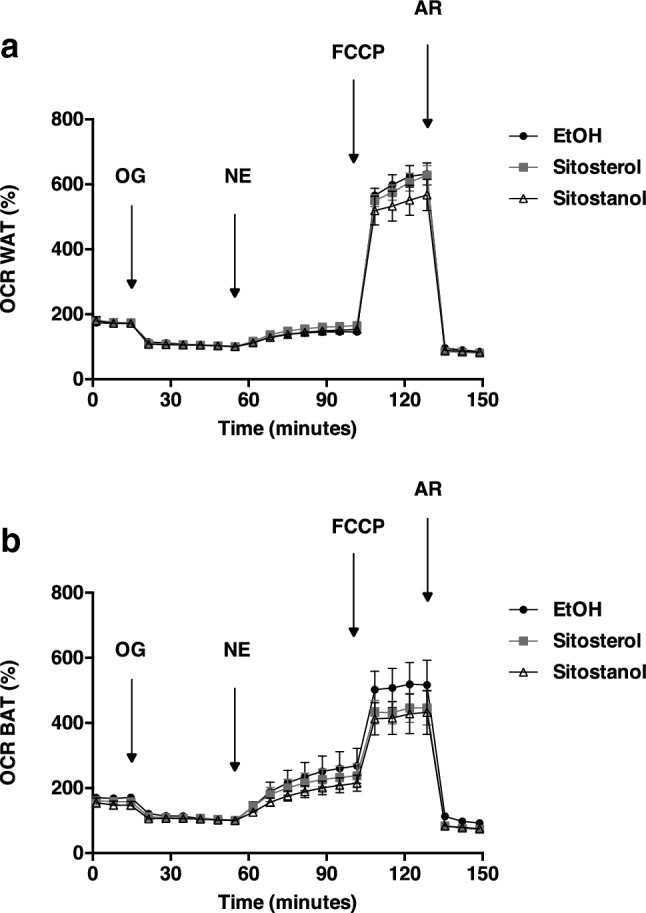
Sitosterol or sitostanol does not alter cellular respiration in cultured human adipocytes. Cellular respiration was measured in cultured adipocytes derived from human WAT (**a**) or BAT (**b**). Adipocytes were incubated for 24 h with sitosterol, sitostanol or vehicle only (EtOH). Cells were exposed to oligomycin (OG), norepinephrine (NE), FCCP and antimycin A + rotenone (AR) at the indicated arrows. Data are expressed as mean ± SEM (*n* = 7 for WAT, *n* = 4 for BAT)

In vitro, we examined the effects of long-term (24 h) incubation of sitosterol or sitostanol on cultured adipocytes derived from human BAT or WAT. In human adipocytes derived from BAT or WAT, neither sitosterol nor sitostanol was able to affect basal oxygen consumption rate (OCR). Adipocytes derived from human BAT showed a clear NE-stimulated increase in OCR, a response that was absent in adipocytes derived from WAT, illustrating the characteristic uncoupling capacity of BAT. However, the addition of sitosterol or sitostanol did not enhance mitochondrial uncoupling capacity as measured by NE-stimulated cellular respiration in adipocytes derived from either human BAT or WAT (Fig. 2a, b).

**Fig. 3:**
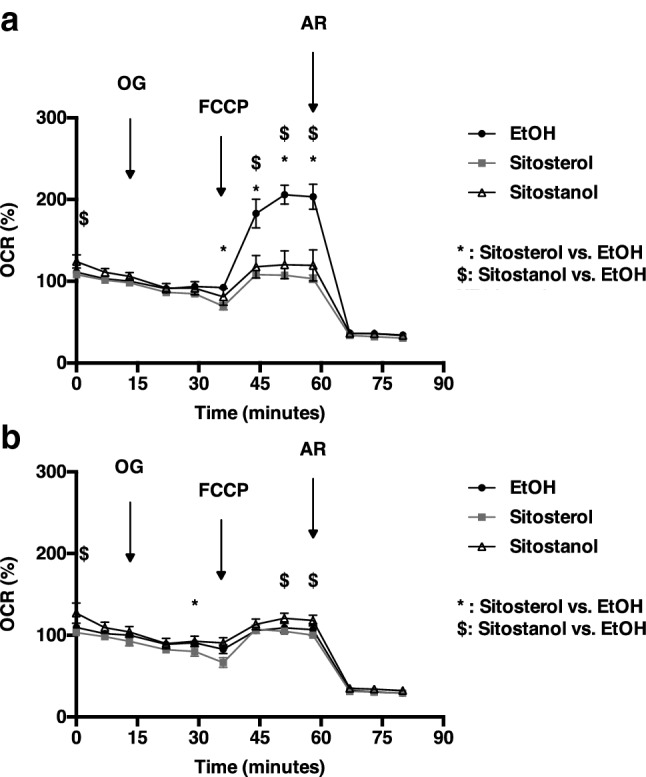
Sitosterol or sitostanol does not change cellular respiration in cultured human hepatocytes (hepG2). Cellular respiration was measured in hepG2 cells in low glucose (5.5 mM, **a**) or high glucose (25 mM, **b**). Cells were incubated with oligomycin (OG), FCCP or antimycin A + rotenone (AR). Data are expressed as mean ± SD (*n* = 8). *p < 0.05 for sitosterol versus control, ^$^*p* < 0.05 for sitostanol versus control

Next, we assessed whether sitosterol or sitostanol can affect cellular respiration in cultured liver cells hepG2 in times of energy deficit, here mimicked by low glucose medium. When hepG2 cells were incubated for 24 h with sitosterol or sitostanol, in contrast to our hypothesis, maximal respiration was significantly decreased when cells were incubated at low glucose concentrations (Fig. 3a, b). When hepG2 cells were tested in high glucose concentration this did not affect cellular respiration in the presence or absence of sitosterol or sitostanol.

**Fig. 4:**
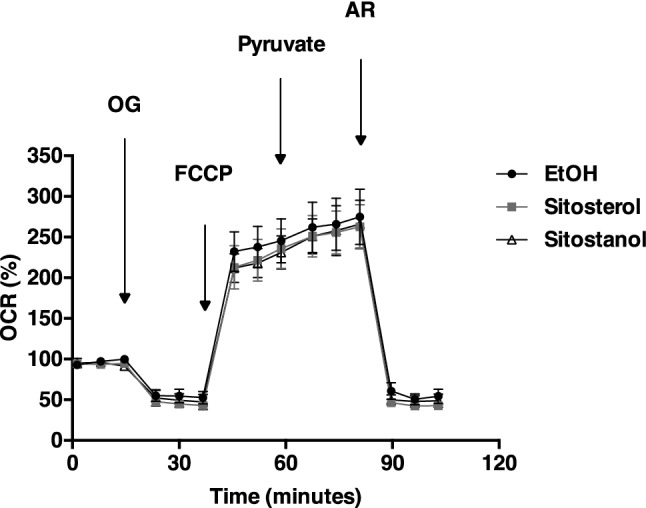
Sitosterol or sitostanol does not alter cellular respiration in cultured human myotubes. Myotubes were exposed to oligomycin (OG), FCCP, pyruvate and antimycin A + rotenone (AR). Data are expressed as mean ± SEM (*n* = 3)

Besides WAT, BAT, and liver, the metabolic syndrome is a major risk factor for the development of type 2 diabetes, in which glucose disposal is compromised the most in skeletal muscle [22]. Therefore, to complete our series of experiments, we examined whether skeletal muscle mitochondrial function was altered, also because human subjects with the metabolic syndrome benefitted from sitosterol or sitostanol-mediated TG reductions. However, our cellular respiration experiments in cultured human myotubes did not show changes following 24-h incubation with sitosterol or sitostanol (Fig. 4).

## Discussion

In the current manuscript, first we demonstrate that mice with elevated plant sterol concentrations show diminished body weight gain. Consuming plant sterols and stanols is an alternative approach to elevate serum concentrations while at the same time lowering serum TG. We hypothesized that elevated mitochondrial activity could link these effects to the elevated plant sterol concentrations. Therefore, we here examined the in vitro effects of sitosterol and sitostanol on mitochondrial function in BAT, WAT, liver, and muscle. Unfortunately, our hypothesis could not be confirmed in the utilized in vitro models for human BAT, WAT, liver or skeletal muscle.

Plant sterols and stanols could potentially affect energy metabolism through mitochondrial metabolism. Having elevated serum plant sterol concentrations, ABCG8 KO mice showed decreased body weight gain compared to control mice consuming the same chow diet (Fig. 1), which could be explained by a change in energy intake. Moreover, Schonewille et al*.* reported that mice fed a HFD-diet enriched with plant sterols or stanols consumed more calories but did not gain more weight compared to control littermates [8]. We proposed that increased BAT activity could not only play a role in lowering TG concentrations but also be responsible for increased energy expenditure resulting in lower body weight gain. Therefore, to evaluate a potential role for BAT in plant sterols and stanols action, we used an in vitro model to examine human BAT activity, which shows similarities to BAT activity in vivo in humans [20, 23]. Although, we did not observe a change in vitro BAT activity, this still would require in vivo experiments in humans to validate our in vitro results. So far, no other in-depth studies on mitochondrial function have been performed on plant sterols and stanols in various organs in parallel, and indeed the few experiments in mitochondria have not always yielded clear results because different cell systems have been used. For example, in isolated mitochondria from the brain, stigmasterol was unable to change ROS production [24]. On the other hand, beta-sitosterol enhanced mitochondrial membrane potential and mitochondrial ATP content in isolated mitochondria from hippocampal neuronal cells [25] and the ATP-stimulating effects of sitosterol have also been observed in H9c2 cardiomyocytes [26]. Not without surprise, high concentrations of stigmasterol can trigger apoptosis in hepG2 [27], which was also observed when using high concentrations of 7beta-hydroxysitosterol in human colon cancer cells [28]. However, in our experiments we have chosen physiologically relevant concentrations without toxic side effects. Various ways exist to measure mitochondrial function, which in the end could introduce variation when measuring mitochondrial function in vitro and/or in vivo. Therefore in the current setting it is of high value that various organs have been examined in parallel using similar experimental setups.

Plant sterols and stanols decrease TGs in mice, which is further associated with decreased secretion of large TG-rich VLDL-1 particles from the liver [8]. In vivo, plant sterols and stanols reach the liver via chylomicrons [29], because of the structural resemblance to cholesterol. In this situation, elevated concentration of plant sterols and stanols in the diet lowered local hepatic inflammation [30],however, experiments on mitochondrial function have not been performed. In contrast, long-term treatment of human subjects with intralipid (which contains plant sterols and stanols) resulted in liver damage [31, 32]. Although it has not been proven that liver damage is causally linked to plant sterols, it is striking that after switching from intralipid to a plant sterol-poor fat emulsion in the parenteral nutrition regimen, liver function is restored [33, 34]. The exact mechanism underlying potential harmful effects of plant sterols when supplied via parenteral routes is unknown,however, disturbed mitochondrial function has been mentioned. Further in-depth experiments on mitochondrial membrane fluidity following sitosterol and sitostanol could provide more information regarding how exactly sitosterol and sitostanol increase ATP content and mitochondrial membrane potential [25] that in the end stimulates whole-body metabolism. This effect on mitochondria is more or less in line with our findings presented here. These findings therefore could be interpreted as being harmful,however, thus far no negative side effects have been reported using plant sterols and/or plant stanols indicating that the human body can counter possible side effects.

To conclude, the TG-lowering potential of plant sterols and stanols is generally accepted. Based on our findings, we here conclude that in vitro mitochondrial function of human BAT, WAT, liver and skeletal muscle can be excluded as a target in the TG-lowering action accomplished by sitosterol or sitostanol. It is therefore most likely that the TG-lowering effects are linked to decreased hepatic VLDL production as proposed earlier.
